# Expression of the Bitter Taste Receptor, T2R38, in Enteroendocrine Cells of the Colonic Mucosa of Overweight/Obese vs. Lean Subjects

**DOI:** 10.1371/journal.pone.0147468

**Published:** 2016-02-11

**Authors:** Rocco Latorre, Jennifer Huynh, Maurizio Mazzoni, Arpana Gupta, Elena Bonora, Paolo Clavenzani, Lin Chang, Emeran A. Mayer, Roberto De Giorgio, Catia Sternini

**Affiliations:** 1 CURE/DDRC, Division of Digestive Diseases, Department Medicine, David Geffen School of Medicine, University of California Los Angeles, Los Angeles, California, United States of America; 2 Department of Neurobiology, David Geffen School of Medicine, University of California Los Angeles, Los Angeles, California, United States of America; 3 Department of Veterinary Medical Science, University of Bologna, Bologna, Italy; 4 Oppenheimer Family Center for Neurobiology of Stress, David Geffen School of Medicine, University of California Los Angeles, Los Angeles, California, United States of America; 5 Department of Medical and Surgical Sciences, University of Bologna, Bologna, Italy; Barnard College, Columbia University, UNITED STATES

## Abstract

Bitter taste receptors (T2Rs) are expressed in the mammalian gastrointestinal mucosa. In the mouse colon, T2R138 is localized to enteroendocrine cells and is upregulated by long-term high fat diet that induces obesity. The aims of this study were to test whether T2R38 expression is altered in overweight/obese (OW/OB) compared to normal weight (NW) subjects and characterize the cell types expressing T2R38, the human counterpart of mouse T2R138, in human colon. Colonic mucosal biopsies were obtained during colonoscopy from 35 healthy subjects (20 OW/OB and 15 NW) and processed for quantitative RT-PCR and immunohistochemistry using antibodies to T2R38, chromogranin A (CgA), glucagon like peptide-1 (GLP-1), cholecystokinin (CCK), or peptide YY (PYY). T2R38 mRNA levels in the colonic mucosa of OW/OB were increased (> 2 fold) compared to NW subjects but did not reach statistical significance (P = 0.06). However, the number of T2R38 immunoreactive (IR) cells was significantly increased in OW/OB vs. NW subjects (P = 0.01) and was significantly correlated with BMI values (r = 0.7557; P = 0.001). In both OW/OB and NW individuals, all T2R38-IR cells contained CgA-IR supporting they are enteroendocrine. In both groups, T2R38-IR colocalized with CCK-, GLP1- or PYY-IR. The overall CgA-IR cell population was comparable in OW/OB and NW individuals. This study shows that T2R38 is expressed in distinct populations of enteroendocrine cells in the human colonic mucosa and supports T2R38 upregulation in OW/OB subjects. T2R38 might mediate host functional responses to increased energy balance and intraluminal changes occurring in obesity, which could involve peptide release from enteroendocrine cells.

## Introduction

The gastrointestinal (GI) tract mucosa detects luminal nutrients and non-nutrients through different effectors and sends information to the nervous system to initiate appropriate functional responses ranging from digestion and absorption to defense mechanisms to protect from outside threat [1–5]. The GI mucosa serves as a sensory organ and expresses a variety of sensory receptors, including short chain fatty acids, bile acid, pathogen-recognition receptors and multiple taste receptors that discriminate palatable from potentially harmful substances [4,6–11]. Sensory receptors are mostly located on enteroendocrine (EEC) cells of the GI mucosa lining, which respond to luminal content by releasing chemical messengers to activate enteric and extrinsic neurons, immune cells or distant targets through the blood stream, and play a critical role in integrating inputs from luminal content and regulating food intake via gut to brain pathways [4,8,12–14].

Taste receptors (TRs) that detect complex tastes such as sweet, savory (umami) and bitter tastes, comprise two major families of G protein coupled receptors, the taste 1 receptors (T1Rs) that function as dimers to detect sweet (T1R2 and T1R3) or umami (T1R1 and T1R3) and the taste 2 receptors, T2Rs that include 25–30 subtypes and detect a large variety of bitter tastants [15–20]. Upon stimulation, TRs interact with specific G protein subunits, such as α-gustducin and other transducers leading to Ca^2+^ increase and transmitter release [15, 21–25]. TRs and associated signaling molecules are found in extra-oral sites, including the digestive system where they are localized to epithelial cells including EEC and brush cells [10,17,25–34]. These findings, together with the observation that tastants increase intracellular Ca^2+^ and induce release of GI peptides from EEC cells *in vitro* and *in vivo*, support the concept that TRs play a role in chemosensing in the gut [17,20,22,28,35,36]. This hypothesis is reinforced by findings that intraluminal tastants induce activation of vagal afferents and food aversion, alter food intake and inhibit gastric emptying [35,37–39]. Moreover, diet manipulations have been shown to alter the expression of TR subtypes and associated signaling molecules further strengthening the concept that taste receptors serve as “sensors” of diet-induced changes in the luminal contents [30,33,40].

In a previous study, we have shown that in mice, a long-term high fat diet inducing obesity results in a selective upregulation in the distal colon of the T2R subtype, T2R138, which is activated by phenylthiocarbamide (PTC), and the signaling molecule α-gustducin. In contrast, a high fat diet does not affect the T2R108, which is activated by denatonium benzoate [33]. Since long-term high fat diet inducing obesity is known to induce gut dysbiosis [41,42] and T2R138 is most abundant in the colon [33], the gut region richest in bacteria, it is likely that T2R138 functions as sensor for subpopulations of intestinal bacteria. Such a sensory role has been shown in the respiratory system where T2R38, the human PTC receptor, is activated by gram-negative bacterial products to induce a protective immune response [43,44].

The aims of this study were to compare the expression of T2R38 in the colonic mucosa of overweight and obese (OW/OB) vs. normal weight (NW) subjects and characterize the types of cells containing T2R38 immunoreactivity (IR) in the human colonic mucosa using samples of colonic biopsies with quantitative RT-PCR, single and double immunofluorescence and confocal microscopy.

## Materials and Methods

### Study participants

Male and female healthy individuals ages 18–55 were recruited primarily by community advertisement. A medical history and physical examination and completion of validated questionnaires measuring GI and non-GI symptoms were performed at the screening visit. Lean (NW) subjects were classified as those with a body mass index (BMI) ≤ 25.0 kg/m^2^ and OW/OB subjects had a BMI ≥ 25.1 kg/m^2^. Study participants did not have personal or family history of GI conditions such as irritable bowel syndrome or other chronic pain conditions. Additional exclusion criteria for all subjects included: infectious or inflammatory disorders, active psychiatric illness as assessed by structured clinical interview for the DSM-IV (Mini-International Neuropsychiatric Interview), or current tobacco or alcohol abuse. All subjects were compensated for participating in the study, and written informed consent was obtained from all subjects. The study was approved by the University of California Los Angeles (UCLA) Institutional Review Board and was conducted in accordance with the institutional guidelines regulating human subjects research.

### Flexible sigmoidoscopies and human colonic biopsies

Colonic mucosal biopsies were taken at 30 cm from the anal verge during a flexible sigmoidoscopy, which was performed in the medical procedures unit. Mucosal samples were collected and immediately snap frozen and stored at -80°C for RNA extraction and qRT-PCR expression analysis or fixed for 2 hours in 4% paraformaldehyde in 0.1 M phosphate buffer, pH 7.4, and paraffin embedded for immunohistochemistry.

### RNA extraction and quantitative Real-Time RT-PCR

Total RNA was isolated from human colonic mucosal biopsies using Qiagen RNeasy Minikit (74104, Qiagen, Valencia, CA) and DNase I treatment was performed to eliminate genomic DNA contamination [33]. RNA quality was assessed by measuring absorbance at 260nm and 280nm (OD260nm/OD280nm > 1.8). We used 2% agarose gel electrophoresis to assess genomic DNA contamination and RNA integrity, which was verified by the presence of two distinct bands that correspond to 18S and 28S rRNA. Complementary DNA was generated using superscript III reverse transcriptase kit (Invitrogen) according to the manufacturer’s instructions on a DNA Thermal Cycler Engine, BIO-RAD. Quantitative real-time reverse transcription polymerase chain reaction (qRT-PCR) was performed using Taqman Gene expression assays for hT2R38 (Applied Biosystem Hs00604294_s1). Standard thermal cycles (40 cycles) for Taqman Gene assays were run on a Mx3000P Real-time PCR DetectionSystem (Stratagene) and data were analyzed with Mx Pro 1000 software. 18S RNA (18S RNA, Applied Biosystem Hs03928990_g1) and β-actin (ACTB, Applied Biosystem Hs4333762T_g1) were used as reference genes and the relative abundance of mRNA expression was calculated using the ΔΔCt method (User Bulletin #2, ABI Prism 7700 Sequence Detection System) [33,45]. Samples were run in duplicates in separate experiments and No-RT and distilled RNAse-free water controls were always included. qRT-PCR products were checked by 4% agarose gel electrophoresis for bands of correct sizes.

### Immunohistochemistry

Serial (5 μm thick) sections from the colonic mucosa of OW/OB (n = 20) and NW (n = 10) subjects were mounted on poly-L-lysine–coated slides and processed for single and double labeling immunofluorescence [30] using antibodies directed against human T2R38, chromogranin A (CgA), or specific markers for EECs subtypes (CCK, GLP-1 and PYY) Table 1. Briefly, sections were deparaffinized through graded ethanols to xylene, rehydrated and heated in sodium citrate buffer (pH 6.0) in a microwave (2 cycles at 600 W, 5 min each) for antigen unmasking. After treatment, slides were incubated in 10% normal donkey serum/0.01 M phosphate buffer saline (PBS) for 1 hour at room temperature in order to reduce non-specific bindings, followed by a combination of primary antibodies made in different species in PBS (overnight, 4°C). Sections were then incubated with a mixture of secondary antibodies (1 hour at room temperature), coverslipped with aqua-poly/mount (Polysciences Inc, Warrington, PA, USA) and stored at -20°C until quantitative analysis was performed. Since the PYY antiserum was raised in rabbit as the T2R38 antiserum, for colocalization studies we utilized the procedure and the appropriate specificity controls previously described by Takechi et al. (2008) to visualize more than one antigen and avoid cross-reactivity of rabbit-generated antisera [46] as in our previously published work [29]. Briefly, sections were sequentially incubated with one antiserum (PYY) at a dilution below the detectability by a standard fluorescently-linked secondary antibody overnight, followed by donkey anti-rabbit IgG linked to biotin. Sections were then incubated with the other antibody (hT2R38) at the optimal dilution for detectability with standard fluorescently-linked secondary antibody overnight, finally they were incubated with a mixture of Texas Red Streptavidin (SA-5006, Vector laboratories, Peterborough, UK) and donkey anti-rabbit IgG-Alexa 488. The levels of detectability of each antiserum with the biotin-streptavidin amplification or the standard fluorescently labeled secondary were previously established for T2R38 and PYY. The sources and dilutions of primary and secondary antibodies are listed in Table 1.

**Table 1:** List of primary and secondary antibodies used in this study with the respective code, source and dilution.

**Primary antisera**	**Species**	**Code**	**Dilution**	**Source**
T2R38	Rabbit	ab65509	1:1000	Abcam
CgA	Goat	Sc-18232	1:200	Santa Cruz
CCK	Mouse	9303	1:800	^#^CURE/DDRC
GLP-1 (7–36)	Mouse	70.113	1:1000	^#^CURE/DDRC
PYY (1–36)	Rabbit	9153	1:2500	^#^CURE/DDRC
**Secondary antisera**		**Code**	**Dilution**	**Supplier**
Donkey anti-Rabbit IgG (H+L), Alexa Fluor^®^ 488 conjugate		A21206	1:1000	Invitrogen
Donkey anti-Goat IgG (H+L), Alexa Fluor^®^ 594 conjugate		A11058	1:600	Invitrogen
Donkey anti-Mouse IgG (H+L), Alexa Fluor^®^ 594 conjugate		A21203	1:1000	Invitrogen
Donkey anti-Rabbit IgG (H+L) biotin conjugate		Ab6801	1:400	Abcam

^#^ CURE/DDRC: CURE Digestive Diseases Research Center, UCLA

The antibody against T2R38 has been well characterized in human circumvallate papillae [47]. Specificity of immunostaining for CgA [10, 30], and CCK [10, 30] has been previously reported in mammals, including humans. Specificity for GLP-1 and PYY antibodies was demonstrated by the lack of immunostaining when the antibodies were pre-adsorbed with an excess of the homologous peptide. In addition, omission of the primary antibody excluded inappropriate binding of the secondary antibody.

### Quantitative assessment of immunolabeled cells

Quantitative analysis of immunolabeled cells was performed to determine the density of T2R38-IR cells in NW vs. OW/OB subjects, and of T2R38-IR cells coexpressing CgA, CCK, GLP-1 or PYY in both groups. Cell counting was performed in a blind manner by two expert operators using a laser scanning confocal microscope (ZEISS 510 Meta, Carl Zeiss Inc, Thornwood, NY) equipped with an appropriate filter to discriminate different fluorescent fluorophores. Since the 40X objective has a field of view = 0.28 mm^2^, we randomly selected to assess 12 fields per sample in order to determine the average number of cells immunoreactive for T2R38, CgA, CCK, GLP-1 or PYY in an area of 3.4 mm^2^. Since we counted immunopositive cells on a 5μm section, a 20μm interval between 4 consecutive sections was selected to avoid cell re-counting and therefore related quantitative biases.

### Statistical analysis

qRT-PCR and cell quantification values were expressed as a mean ± standard error of the mean (SEM). Differences in mRNA level and cell number between the two groups of interest (OW/OB vs. NW) were analyzed using the unpaired Student’s *t*-test, whereas for comparison among different groups of EEC cells in OW/OB and NW subjects we used one-way ANOVA followed by the Bonferroni test (Graph Prism 5, GraphPad Softwere, Inc., La Jolla, CA, USA). P<0.05 was considered significant. To determine whether T2R38 expression correlated with the BMI of our subjects, we did a correlation analysis between the T2R38 mRNA levels or T2R38-IR cell numbers and the BMI values of the subjects investigated using D’Agostino and Pearson normality test followed by Spearman rank correlation test (Graph Prism 5, GraphPad Softwere, Inc., La Jolla, CA, USA).

## Results

### Clinical characteristics of study participants

A total of 35 healthy volunteers participated in the study and included 20 OW/OB subjects and 15 lean subjects. The 15 NW subjects were comprised of 7 men and 8 women with a mean age of 34.8 ± 3.0 years (range 22–55) and had a mean BMI of 20 ± 0.5 kg/m^2^. Of the 20 OW/OB subjects, there were 11 men and 9 women with a mean age of 36.5 ± 2.8 years (range 20–55) and mean BMI of 32 ± 0.7 kg/m^2^. Samples from these subjects (35 total) were used for qRT-PCR (11 NW and 14 OW/OB) and/or for immunohistochemistry (10 NW and 20 OW/OB).

### hT2R38 mRNA levels and T2R38-IR cells density in the colonic mucosa of normal weight and overweight/obese subjects

**Fig 1:**
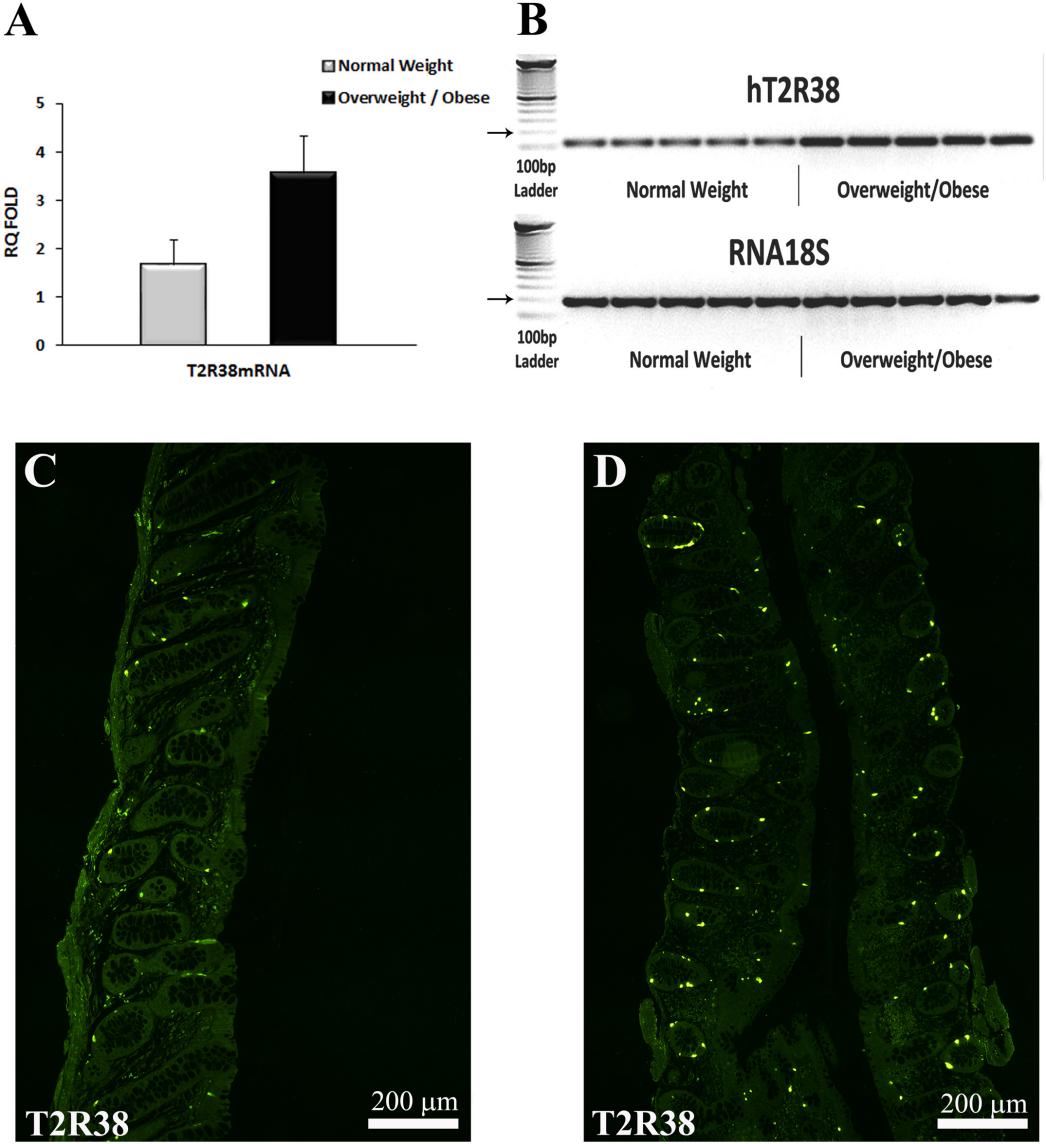
T2R38 mRNA levels and T2R38-IR cell in colonic mucosal biopsies of NW and OW/OB subjects. (A) T2R38 mRNA levels in NW and OW/OB subjects analyzed by means of qRT-PCR and normalized to 18S RNA levels. Each cDNA sample was amplified in duplicate and all data are expressed as the mean ± SEM. The levels of T2R38 mRNA were markedly, but not significantly (P = 0.06) increased in OW/OB compared to NW subjects. (B) Single bands of the predicted size (116 bp for T2R38) were found in colonic samples of OW/OB and NW subjects. RNA 18S (187 bp; arrow) served as reference gene. The left column shows the ladder. (C and D) Confocal images of T2R38 immunoreactivity (IR) in the colonic glands in NW (C) and OW/OB (D) subjects. Note the markedly higher density of T2R38-IR cells in OW/OB vs. NW subjects. Calibration bar: 200 μm.

Since the levels of T2R38 mRNA in OW and OB subjects were comparable, they were grouped together. There was a marked increase (>2 fold) in the level of expression of T2R38 mRNA in OW/OB compared to NW subjects (3.56 ± 0.8 vs. 1.68 ± 0.5, respectively; n = 11 NW and n = 14 OW/OB) (Fig 1A), which did not reach statistical significance (P *=* 0.06).

T2R38-IR was localized to cells located in the epithelial lining and throughout the glandular epithelium of the descending colon in NW and OW/OB subjects (Fig 1C and 1D). T2R38-IR cells were significantly more abundant in the OW/OB than in NW individuals (P = 0.01) Table 2.

**Table 2:** Quantification of T2R38-IR and CgA-IR cells in NW and OW/OB individuals.

Subjects	T2R38-IR cells	CgA-IR cells	%T2R38-/CgA-IR cells
**Normal Weight n = 10**	55.9± 7.9	138 ± 5.6	40.9±3.1
**Overweight/Obese n = 20**	124.9 ± 16.0**	146 ± 2.7	86.4±1.7***

Values represent the mean ± SEM.

** *P* < 0.01,

*** *P* <0.001 vs. normal weight group.

**Fig 2:**
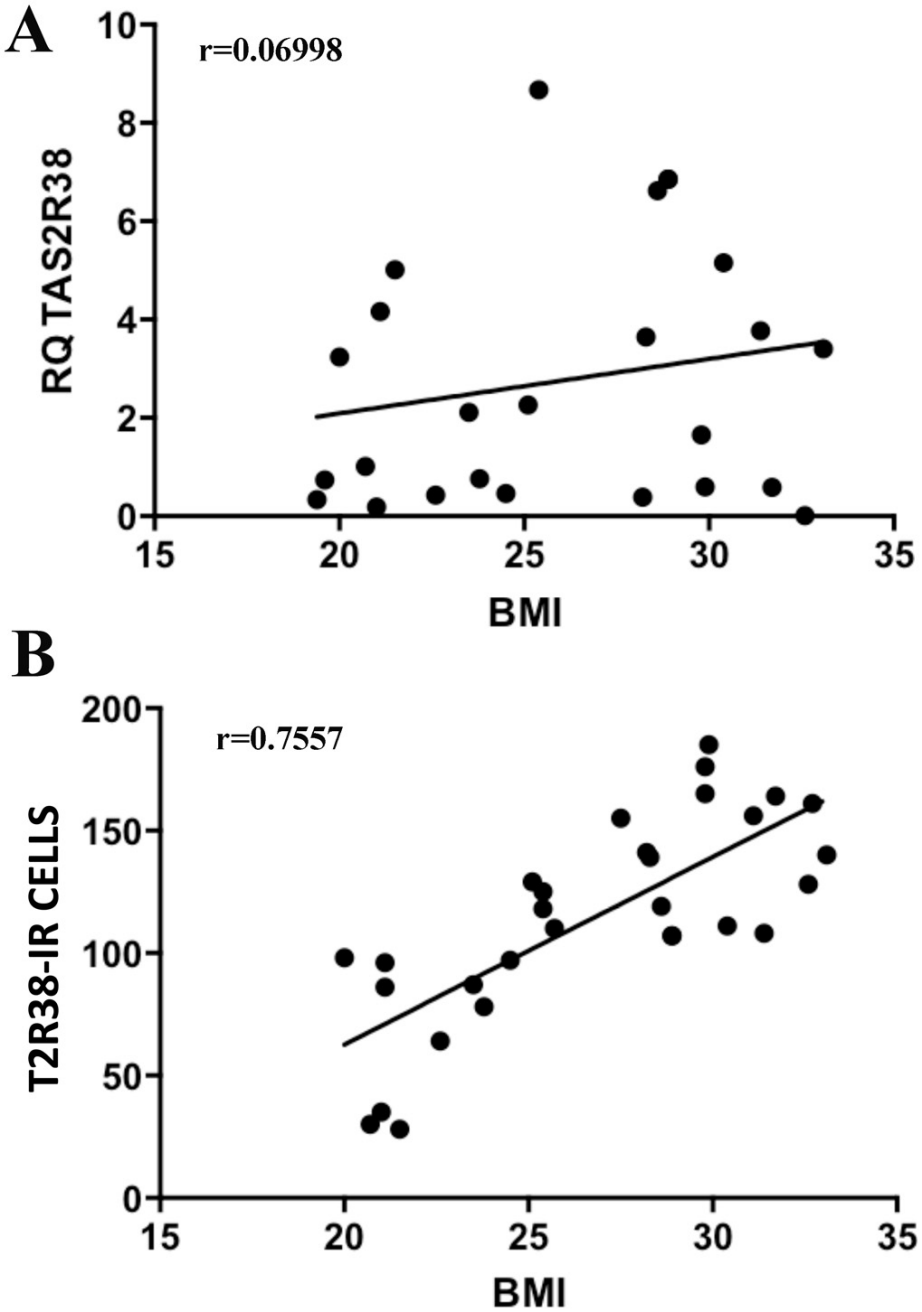
Graphical representations of Spearman Correlation (r) test. Spearman correlation test was performed between T2R38 expression and BMI in the subjects included in this study. There was no significant correlation between T2R38 mRNA levels and BMI values (A) (r = 0.06998; P = 0.7694), but there was a highly significant and positive correlation between the density of T2R38-IR cells and BMI values (B) (r = 0.7557; P = 0.001).

There was a highly significant positive correlation between the density of T2R38-IR cells and BMI values (P = 0.001), but not between T2R38 mRNA levels and BMI (Fig 2).

### Characterization of T2R38-IR cells in colonic mucosa of NW and OW/OB subjects

**Fig 3:**
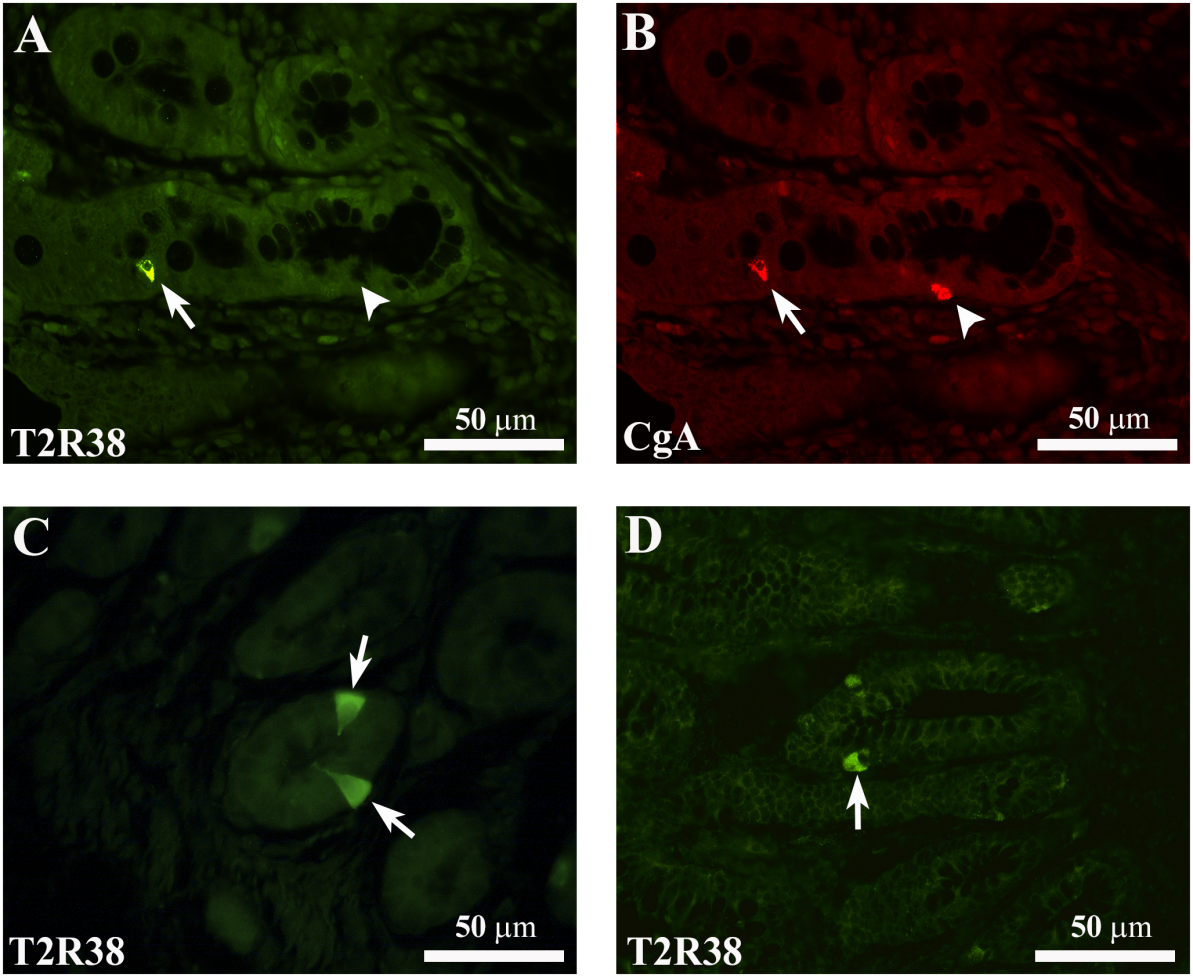
Representative confocal images of T2R38- and CgA-IR cells. (A) Shows a T2R38-IR cell (arrow) which is immunoreactive for chromogranin A (CgA)-IR (B, arrow) in the colonic mucosa of a NW subject. The arrowheads in A and B indicate a CgA-IR cell not containing T2R38-IR. C and D show the different types of morphology of T2R38-IR cells. Arrows in C point to an “open-type” EEC cell with T2R38-IR, while the arrow in D points to a T2R38-IR cell displaying characteristics of “closed-type” EEC cells. Images in C and D are from colonic mucosa of OW/OB subjects. Both T2R38-IR types of cells were observed also in the colonic mucosa of NW subjects (not shown). Calibration bar: 50μm.

All the T2R38-IR cells co-expressed CgA, while not all CgA-IR cells co-expressed T2R38-IR Table 2 (Fig 3A and 3B) in either NW or OW/OB subjects. T2R38-IR cells had an elongated or pear shape with a homogenously labeled cytoplasm and some cells were characterized by cytoplasmic prolongations extending up to the endoluminal surface, consistent with an 'open type' morphology, while others exhibited a 'close type' profile with rounded shape cells and were confined to the basal lamina (Fig 3C and 3D) [5,14].

**Fig 4:**
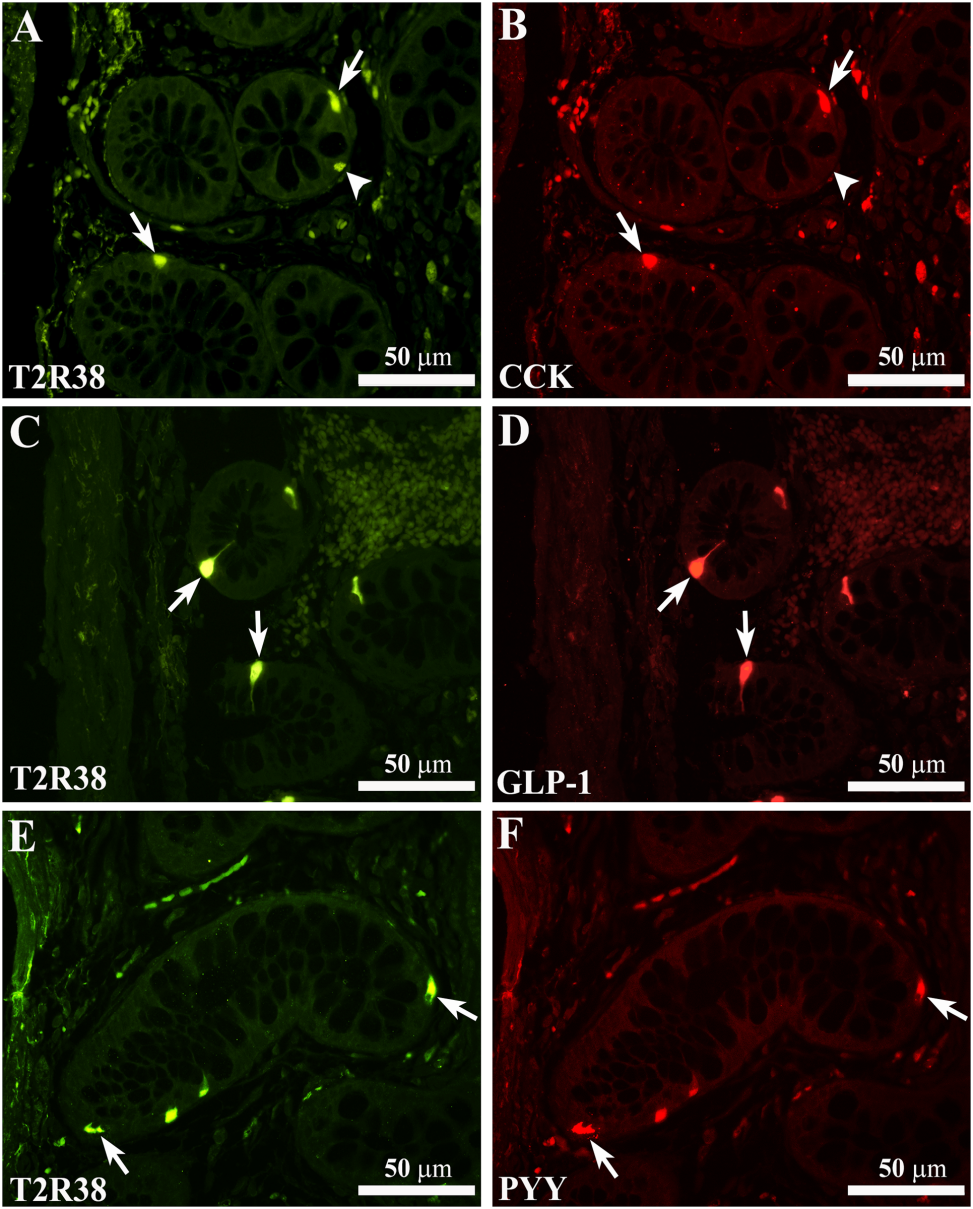
Colocalization of T2R38-IR with CCK-, GLP-1- or PYY-IR immunoreactivity. Confocal images showing T2R38-IR cells (A, C and E, arrows) containing immunoreactivity for CCK (B), GLP-1 (D) or PYY (F). The arrowheads in A and B indicate a T2R38-IR cell not containing CCK-IR. Calibration bar: 50μm.

T2R38-IR colocalized with immunoreactivity for CCK, GLP-1 or PYY (Fig 4A–4F), peptides that are released by different nutrients, affect digestive functions and act as satiety signals [2,48]. T2R38/CCK-, GLP-1- or PYY-IR cells showed either elongated or pear-like shape (Fig 4A–4F).

**Fig 5:**
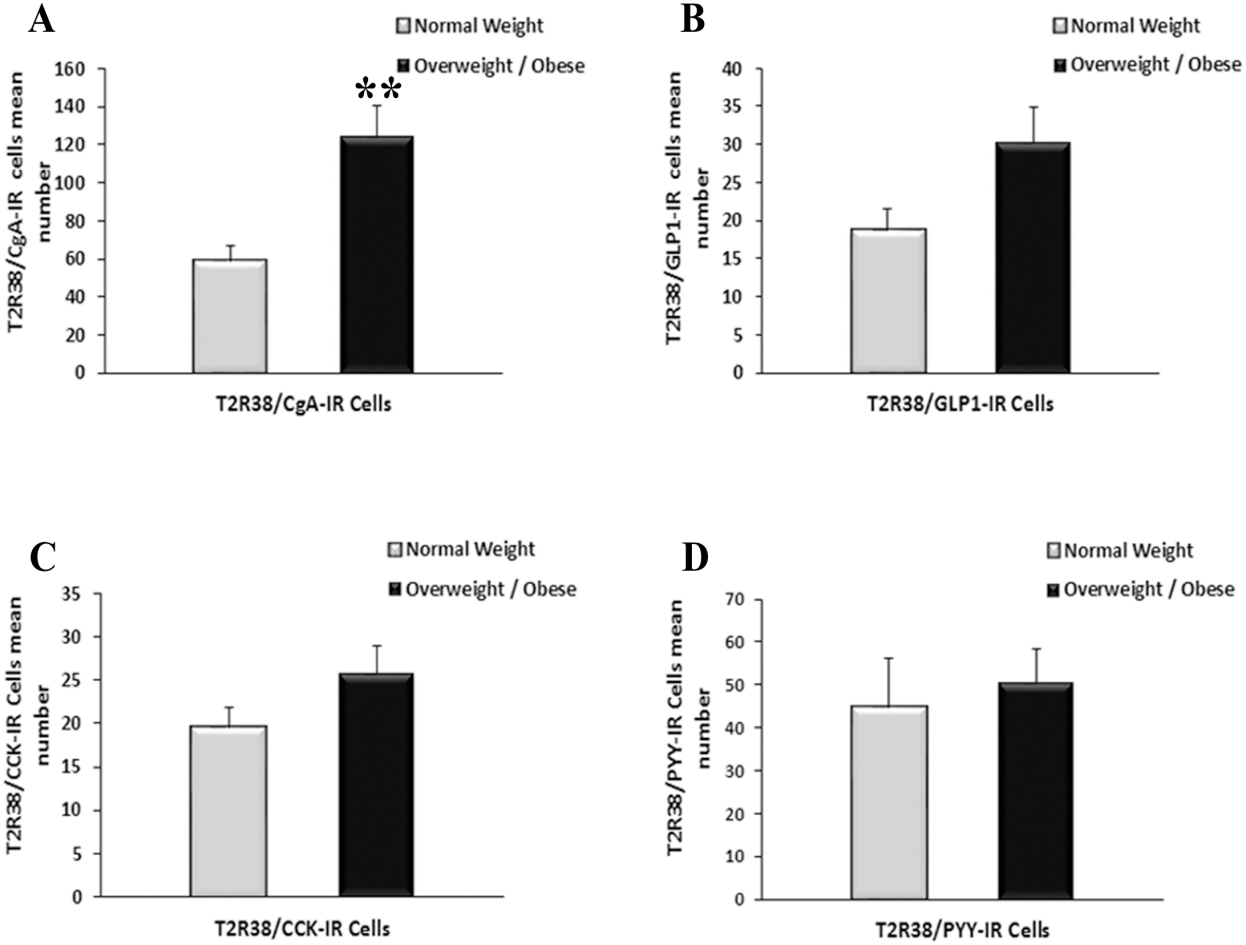
Density of T2R38 subpopulations of EEC cells in NW and OW/OB subjects. (A) Density of T2R38/CgA-IR cells in NW compared to OW/OB subjects. (B-D) Density of T2R38/GLP-1-IR (B), T2R38/CCK-IR (C) and T2R38/PYY-IR (D) cells in NW and OW/OB subjects. All data are expressed as mean ± SEM. ** indicate a statistically significant difference, *P* = 0.01 between NW and OW/OB groups.

T2R38/CgA-IR cells are significantly increased in OW/OB vs. NW subjects, whereas the overall CgA cells population (T2R38 positive and T2R38 negative) was similar in OW/OB and NW subjects Table 2 (Fig 5A). The percentage of T2R38/CgA-IR cells in the OW/OB group was more than doubled compared to NW subjects as shown in Table 2 (P<0.001). There was no significant difference in the number of T2R38/GLP1-IR cells (30.3 ± 4.6 vs. 18.7 ± 2.8), T2R38/CCK-IR cells (25.7 ± 3.2 vs. 19.6 ± 2.2) or T2R38/PYY-IR cells (50.7 ± 7.9 vs. 44.8 ± 11.4) in OW/OB vs. NW individuals (Fig 5B–5D). Similarly, there was no significant difference between the number of CCK-IR, GLP-1-IR and PYY-IR cells in OW/OB compared to NW as illustrated in Table 3. The highest T2R38-IR expression was in the CCK population in both the OW/OB and NW group, and the percentage of T2R38/CCK-IR cells was significantly higher in OW/OB vs. NW subjects, whereas there were no significant differences between the percentages of the T2R38/GLP1-IR or T2R38/PYY-IR in OW/OB vs. NW groups

**Table 3:** T2R38-IR expression in CCK, GLP-1 and PYY populations in normal and overweight/obese subjects.

Subjects	CCK-IR cells	%T2R38/ CCK-IR cells	GLP1-IR cells	%T2R38/ GLP1-IR cells	PYY-IR cells	%T2R38/ PYY-IR cells
**NW n = 10**	22.9±2.4	85.7±1.8*	49.8±7.8	45.1±8.7	59±13	67.1±8.7
**OW/OB n = 20**	27.2±3.1	93.1±2.9*	59.8±6.7	54.1±5.6	88.5±10.1	56.7±5.4

Values represent the mean±SEM of the average number of cells and the % of each EEC population expressing T2R38-IR.

* *P*< 0.03 between %T2R38/CCK-IR cells in OW/OB vs. NW subjects.

## Discussion

This study shows that the bitter taste receptor subtype, T2R38 is expressed by the human colonic mucosa, where it is localized to distinct types of EEC cells, including CCK, GLP-1, and PYY cells. We show for the first time that T2R38 is upregulated in overweight and obese subjects as indicated by the marked and significant increase in number of T2R38-IR cells without changes in the overall density of EEC cells in the colonic mucosa of OW/OB vs. NW subjects. In the OW/OB group, there was also a trend in T2R38 mRNA increase (~2 fold compared to the NW group), but the difference between the two groups did not reach statistical significance. This could be explained by differences between T2R38 transcription and translation or by the smaller number of samples from OW/OB subjects utilized for qRT-PCR analysis compared to those used for the immunohistochemical analysis (14 and 20, respectively). T2R38 is confined to EEC cells in the human colonic mucosa, since all epithelial cells identified with T2R38 immunostaining were also immunoreactive for the EEC cell generalized marker, CgA. This differs from the mouse, where the colocalization between T2R138, the mouse counterpart of the human T2R38, and CgA was not complete indicating that other cell types express this T2R138 subtype [33]. This is in line with the distribution of α-gustducin, the major taste receptor signaling molecule, which has been found in EEC and non-EEC cells in the mouse GI tract [26,32,49], whereas it has been localized only in EEC cells in the human colonic mucosa [10]. EEC cells are regarded as primary chemoreceptors that respond to luminal constituents by releasing secretory products that activate neuronal pathways, nearby cells or distant targets to induce functional responses [2,3,5]. The presence of T2R38-IR in both EEC ‘open cells’ with extensions reaching the luminal surface where they can detect luminal content, and EEC ‘closed cells’ that do not reach the lumen but can be regulated by luminal content indirectly through neural and humoral mechanisms [2,50], is consonant with T2R38 acting as sensor of luminal contents. The increased expression of T2R38 in the mucosa of OW/OB compared to NW subjects suggests a modulation of this TR in the context of obesity, and possibly alterations in dietary composition.

T2R38 expression was most abundant in EEC cells producing CCK ranging from ~85% of the CCK-IR cells in NW to ~93% in OW/OB individuals. CCK is a gut peptide released by the breakdown of proteins and fats, which modulates gastrointestinal secretion and motility, triggers digestive enzyme release and induces satiation via vagal afferent neurons [2,51]. T2R38 expression is also abundant in cells producing GLP-1 and PYY ranging from ~45% to >65% of GLP-1- and PYY-IR cells, respectively. GLP-1 and PYY are peptides that are released in response to different nutrients such as fats and carbohydrates. GLP-1 and PYY are incretins involved in nutrient absorption and energy storage and implicated in the pathogenesis of metabolic disorders, including obesity and type 2 diabetes [1,52,53]. GLP-1 and PYY also serve as satiety signals and mediate an aversive food response similarly to what observed by intraluminal bitter stimuli [37,38,54,55]. The abundant expression of T2R38-IR in different EEC subpopulations is consistent with the involvement of this receptor in the biological functions mediated by different peptides released upon activation of these cells. T2R38 might regulate energy balance, food intake, absorption and satiety through peptide release in response to intraluminal changes in both NW and OW/OB individuals. Indeed, bitter tastants, including T2R38 selective ligand, PTC, induce CCK and GLP-1 release from EEC cells in vitro and in vivo [9,22,28] as well as PYY release (unpublished data). Intraluminally administered bitter ligands, including PTC, have been shown to activate vagal afferents presumably through the release of bioactive peptides including PYY and CCK resulting in modulation of ingestive behavior, motility and secretion [37–39]. The significant upregulation of T2R38 in the overall EEC cell population might represent adaptation of the GI mucosa to intraluminal changes induced by increased food intake and obesity. It is now recognized that EEC cells have complex phenotypes and can produce different peptides, which may affect each other when released upon stimulation [56]. T2R38 activation could affect body homeostasis through the release of multiple peptides including CCK, GLP-1, and PYY but also gastric inhibitory peptide and GLP-2 that can be produced by the same cells containing GLP1 and PYY [56]. In animal models, we have previously shown that different types of diets altered the expression of T2R subtypes and taste-associated molecules in different regions of the gut [30,33], and that a long-term high fat diet inducing obesity resulted in T2R138 upregulation in the mouse colonic mucosa, where T2R138-IR was localized to EEC cells [33]. Whereas we do not know the dietary composition in the subjects included in the present study, OW/OB subjects had a positive energy balance as indicated by increased BMI presumably due to increased food consumption compared to NW individuals, which is likely to result in changes in the luminal content. This is supported by the highly significant and positive correlation of T2R38-IR cell density and the BMI values. Whether there are pre-existing differences in the number of T2R38/EEC cells in individuals who become overweight or obese cannot be answered by our findings. T2R138 (the rodent equivalent of T2R38 in human) was upregulated in the colon mucosa of mice fed long-term high fat diet but not in their littermates fed regular diet [33], suggesting that the changes in T2R138 expression in these animals were diet-induced. It is reasonable to hypothesize that T2R38 upregulation in the colonic mucosa of the OW/OB subjects is also induced by the excess of fat intake resulting in excess weight. Altered expression of taste molecules, including increased expression of gustatory signaling elements and a decrease in T1R3, the sweet-umami receptor [17,27], has also been reported in the gastric mucosa of morbidly obese patients compared to controls [40]. All together, these findings support the involvement of taste elements in chemosensing in the GI tract.

Bitter taste has evolved as a central warning signal against harmful substances and elicits defensive responses. Several lines of evidence support that extra-oral T2Rs might serve as defense mechanisms against external or internal threats. In the gut, bitter tastants induce conditioned flavor avoidance, inhibit food intake and gastric emptying, modulate gut efflux membrane transporters and increase intestinal chloride secretion [28,35–38,57], mechanisms aimed at reducing the contact with and exposure to harmful substances. The GI lumen contains a large variety of substances ranging from nutrients and enzymatic breakdown of ingested food, food-born contaminants, toxins, microbial products and bacteria, many of which could pose a threat [4]. It is known that obesity is associated with changes in luminal microbial composition (such as an increased Firmicutes to Bacteriodetes ratio) resulting in low grade chronic inflammation in the gut and other organs, and is likely to represent the initial stage of metabolic disarray [41,42,58]. The human colon is one of the gut regions with the highest microbial density, which is estimated to be around 10^11^−10^12^ per gram of contents containing up to a thousand species [59]. Since bacterial populations are altered by obesity-inducing diets, one can hypothesize that T2R38 senses bacteria or their products, which induce functional responses mediated by peptide release. Indeed, in a pilot study, we found that antibiotic treatment that has been shown to reduce diet-induced gut dysbiosis, reverses diet-induced T2R138 upegulation, supporting the concept that this bitter taste receptor interacts with luminal bacteria (unpublished data). This is in line with findings in the respiratory system, where T2R38 has been shown to detect bacterial signals such as quorum sensing molecules that regulate bacteria-bacteria and bacteria-host communication and initiate an immune response and antimicrobial program to protect from pathogens [43,44,60]. Interestingly, T2R38 is characterized by polymorphisms that determine the ability to detect bitterness, which might influence food intake, but also affect individual susceptibility to respiratory infections and are associated with changes in eating behaviours [44,61,62]. Overweight and obesity defined as abnormal or excessive fat accumulation directly related to imbalance between caloric intake and energy expenditures affect millions of people worldwide and represent an important burden to the society because of the impact on morbidity and mortality due to metabolic disarray [63,64]. The identification of sensory receptors detecting changes in luminal contents in diet-induced weight increase represents an important step toward the elucidation of the molecular events underlying intraluminal chemosensing and ultimately the discovery of new therapeutic approaches for obesity.
